# Despite genetic isolation in sympatry, post-copulatory reproductive barriers have not evolved between bat- and human-associated common bedbugs (*Cimex lectularius* L.)

**DOI:** 10.1186/s12983-023-00514-y

**Published:** 2023-11-10

**Authors:** Markéta Sasínková, Ondřej Balvín, Jana Vandrovcová, Christian Massino, Alfons R. Weig, Klaus Reinhardt, Oliver Otti, Tomáš Bartonička

**Affiliations:** 1 Department of Ecology, Faculty of Environmental Sciences, Czech University of Life Sciences Prague, Kamýcká 129, 165 21 Prague 6, Czech Republic; 2 Department of Botany and Zoology, Faculty of Science, Masaryk University , Kotlářská 2, 611 37 Brno, Czech Republic; 3 Applied Zoology, Department of Biology, Technische Universität Dresden, 01062 Dresden, Germany; 4 Genomics and Bioinformatics, University of Bayreuth, Universitätsstrasse 30, 95440 Bayreuth, Germany; 5 Animal Population Ecology, Animal Ecology I, University of Bayreuth, Universitätsstrasse 30, 95440 Bayreuth, Germany

**Keywords:** Host fidelity, Host adaptation, Ecological speciation, Sperm storage

## Abstract

**Background:**

The common bedbug *Cimex lectularius* is a widespread ectoparasite on humans and bats. Two genetically isolated lineages, parasitizing either human (HL) or bat (BL) hosts, have been suggested to differentiate because of their distinct ecology. The distribution range of BL is within that of HL and bedbugs live mostly on synanthropic bat hosts. This sympatric co-occurrence predicts strong reproductive isolation at the post-copulatory level.

**Results:**

We tested the post-copulatory barrier in three BL and three HL populations in reciprocal crosses, using a common-garden blood diet that was novel to both lineages. We excluded pre-copulation isolation mechanisms and studied egg-laying rates after a single mating until the depletion of sperm, and the fitness of the resulting offspring. We found a higher sperm storage capability in BL, likely reflecting the different seasonal availability of HL and BL hosts. We also observed a notable variation in sperm function at the population level within lineages and significant differences in fecundity and offspring fitness between lineages. However, no difference in egg numbers or offspring fitness was observed between within- and between-lineage crosses.

**Conclusions:**

Differences in sperm storage or egg-laying rates between HL and BL that we found did not affect reproductive isolation. Neither did the population-specific variation in sperm function. Overall, our results show no post-copulatory reproductive isolation between the lineages. How genetic differentiation in sympatry is maintained in the absence of a post-copulatory barrier between BL and HL remains to be investigated.

**Supplementary Information:**

The online version contains supplementary material available at 10.1186/s12983-023-00514-y.

## Background

Speciation, the ultimate source of biodiversity, is the evolutionary process in which populations diverge genetically to become distinct species. One precondition for speciation is reproductive isolation, defined as any kind of behavioural, physiological, or other barriers that reduce fitness in crosses between, compared to within, populations. Reproductive barriers exist at the pre- and post-zygotic levels. Prezygotic processes can be divided into pre- and post-mating mechanisms. Pre-mating mechanisms have been studied excessively because they are believed to be the main mechanism to prevent investment into inferior hybrid zygotes [1–5]. Post-mating prezygotic isolation encompasses preferential within- over between-population fertilization once copulation occurred [6]. It occurs in the form of gametic incompatibility [7] in a variety of taxa ranging from sea urchins [5] to fish [8] and insects [9]. Post-mating prezygotic isolation may also occur as population-specific sperm precedence [4], known, for example, from *Drosophila* [10] and other insects [11–13].

Reproductive barriers drive reproductive isolation to speciation only if the reproductive barriers have a genetic basis [2, 4]. The genetic basis, known as Dobzhansky–Muller incompatibilities [2], can evolve in three major ways, either as a neutral or an adaptive process. i) Over time, neutral genetic divergence accumulates and at some stage reduces genomic compatibility between populations, i.e., reproductive isolation is a by-product of divergence. Many speciation processes seem to work according to this scenario, such as isolation-by-distance or the ring species [2]. ii) Genetic divergence between populations may be caused by sexual selection [10, 14–17] and—not necessarily entirely dissimilar—iii) by divergent natural selection in ecologically distinct populations. Gene flow barriers evolve between populations as a result of ecologically divergent selection for both allopatric and sympatric divergence [3, 4, 18, 19]. Examples include population differences in herbivores adapting to different host plants [3, 4], benthic fish to different diets [18], and parasites to different hosts (alloxenic speciation), [16, 20].

Common bedbugs *Cimex lectularius* Linnaeus, 1758 are obligate blood-feeding, wingless ectoparasites that feed on either humans or bats. The human-associated lineage (HL) and the bat-associated lineage (BL) probably split more than 200,000 years ago [21] and have remained genetically isolated [22]. With the development of stable human settlements around 10,000 years ago [23] and BL populations following the synanthropic lifestyle of several bat species [24, 25], BL and HL became sympatric. Therefore, while encounters of HL and BL can be expected, we assume that the lack of genetic evidence of their contact suggests that the genetic isolation of the two lineages may be driven by their host specialization.

Morphological differences exist between BL and HL, some of which might reflect adaptation to their hosts [21]. Lower fecundity and survival of HL individuals reared on bat blood compared to human blood [26], would also point to adaptations. BL individuals have no access to their (migrating) host between autumn and spring, whereas HL have continuous host access. Because feeding is closely linked to mating in bedbugs [27], HL females can thus obtain a constant sperm supply via continuous mating, while BL females are predicted to invest more in long-term sperm storage. Given that sperm metabolism can evolve in response to female mating rate in insects [28], we might also expect ecologically driven differences in sperm biology between the two lineages. Differences in sperm biology may contribute to reproductive isolation between BL and HL.

Reproductive isolation between BL and HL has been studied in the context of their clear ecological, morphological, and genetic separation. The results appear to be partially inconsistent. Wawrocka et al*.* [29] found strong reproductive isolation because the between-lineage crosses failed to produce any eggs. Using a subset of the same BL populations several generations later, Křemenová et al*.* [30] showed that BL males are compatible with HL females. However, Křemenová et al*.* [30] did not test the compatibility of BL females with HL males. Subsequent studies, using a different set of one BL and one HL [31] or two BL and three HL populations [32], found full compatibility of between-lineage crosses in both directions. However, in these studies, crosses using individual populations were replicated only twice [32] or female fecundity was evaluated for only 6 days [31, 32], whereas females can lay eggs after one mating and regular feeding for up to ten weeks [33–36]. Differential sperm use during storage [10, 37, 38] might alter the reproductive output of females and so contribute to explain the inconsistencies between studies. For example, ecological speciation would predict more eggs being fertilized, and therefore more sperm being used, in within-lineage crosses compared to between-lineage crosses. Then, sperm numbers would decrease more rapidly over time and any detrimental fecundity effects would appear only late in the reproductive cycle, certainly not within six days. DeVries et al. [32] successfully bred F2 progeny from the F1 generation of BL and HL crosses, but we know little about the success of the F1 generation, such as offspring size or survival. Finally, and in contrast to other studies, Křemenová et al. [30] manipulated diet separate from lineage and measured the ability of HL females to store sperm from HL and BL males: HL sperm performed better when males were fed on bat blood than on human blood.

In this paper, we assess the effect of genetic differentiation on reproductive isolation by measuring the gametic compatibility and hybrid fitness of HL and BL. We used three population replicates for both HL and BL, one of which has been used previously [29, 32] to determine the relative effect of genetic distance on female fitness and offspring size and survival. In this way, we tested the hypothesis that genetic isolation in sympatry is at least partially based on post-copulatory isolation. Based on a set of nine microsatellite loci, all populations used are clearly distinct from each other (F_ST_ > 0.24). We measured female fecundity after a single controlled mating for ten weeks. To account for possible effects of BL and HL genome divergence on the progeny, we tested the fitness of the offspring between and within HL and BL.

## Results

### Genetic divergence between populations

All populations showed lower levels of heterozygosity than expected (Additional file 2: Table S1), and all loci deviated from Hardy–Weinberg equilibrium (Additional file 2: Table S2). The deviation is in line with previous microsatellite studies on the common bed bug [22, 39] and can be well expected for an insect with such a specific lifestyle. The presence of potential null alleles was suggested only for one locus (BB454_20), which was kept in further analyses.

The pairwise F_ST_ values between populations were rather large, exceeding 0.350 in all pairs except for Hanušovice and Raškov for which F_ST_ reached 0.245 (Additional file 2: Table S3), also suggesting that the populations are distant and largely separate. This is further confirmed by AMOVA showing that significant variation is explained by population, no matter if the lineages are analyzed together or separately (Additional file 2: Table S4), and by the relatively large numbers of private alleles for each population (Additional file 2: Table S1).

### Reproductive isolation between host lineages

A similar number of females laid no eggs within- and between-lineage crosses (Fisher exact test: *P* = 0.61). Significantly more HL females perished during egg-laying than BL females (Fisher exact test: *P* = 0.03). From the total of 369 females, 43 laid fertilized eggs for more than 10 weeks; eggs laid after the tenth week were not included in the analyses.

#### Number of eggs

**Fig. 1:**
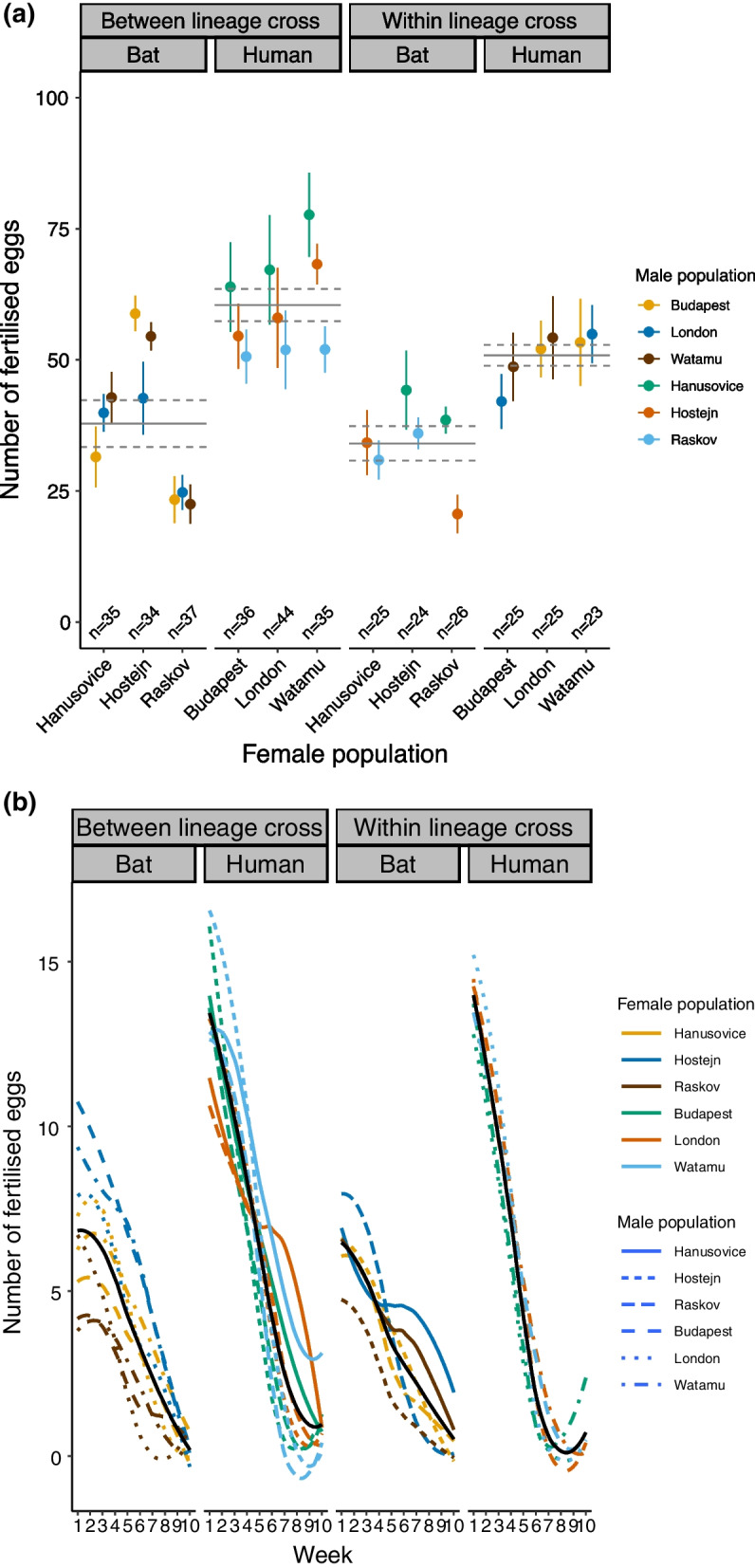
The number of fertilized eggs laid over ten weeks for each population cross. **a** The total number of fertilized eggs. Original host of the female population is indicated at the top of the plot. The colored symbols show population means, the lines show means for lineage crosses, and the dashed lines represent one standard error. Female populations are shown on the x-axis and the colors represent the male populations. Error bars represent one standard error. **b** The number of eggs laid per week, with different line types representing the male populations and colors representing the female populations. The black solid lines show the mean egg-laying curve for each lineage cross, and grey shading represents 95% confidence intervals

Independent of lineage, the number of eggs did not differ between the within-lineage crosses and between-lineage crosses (LME lineage cross type x female lineage: fertilized eggs—F_1,26.82_ = 1.907, *P* = 0.179; total eggs—F_1,26.83_ = 1.183, *P* = 0.286) (Fig. 1a, Additional file 1: Fig. S1). Similarly, egg counts did not differ between the lineage cross types (LME: fertilized eggs—F_1,26.34_ = 0.096, *P* = 0.759; total eggs—F_1,26.34_ = 0.106, *P* = 0.748). However, HL females laid significantly more eggs than BL females (LME: fertilized eggs—F_1,25.67_ = 30.605, *P* < 0.0001; total eggs—F_1,25.67_ = 43.884, *P* < 0.0001) (Fig. 1a, Additional file 1: Fig. S1).

The three-way interaction of the week x female lineage x lineage cross type was significant for the number of fertilized eggs (LME: F_1,3308.2_ = 15.374, *P* < 0.0001). Neither the female lineage x lineage cross type interaction (LME: F_1,40.4_ = 1.472, *P* = 0.232) nor the week x lineage cross type interaction were significant for the number of fertilized eggs laid by the females (LME: F_1,3308.5_ = 2.050, *P* = 0.152). HL females laid more fertilized eggs at the beginning of the experiment, but their fertilized egg-laying rate decreased faster over time than in BL females (LME: week x female lineage: F_1,3315.0_ = 368.309, *P* < 0.00001, Fig. 1b). The number of fertilized eggs laid per week decreased significantly over time in all crosses (LME: F_1,3329.3_ = 3222.324, *P* < 0.0001).

The proportion of unfertilized eggs did not show a significant interaction term between lineage cross type and female lineage (GLME with binomial distribution: X^2^ = 0.955, df = 1, *P* = 0.329), nor did it differ between the lineage cross types (GLME with binomial distribution: X^2^ = 0.206, df = 1, *P* = 0.650). HL females laid a significantly higher proportion of unfertilized eggs than BL females (GLME with binomial distribution: X^2^ = 10.134, df = 1, *P* = 0.0015, Additional file 1: Fig. S2).

#### The onset of infertility

**Fig. 2:**
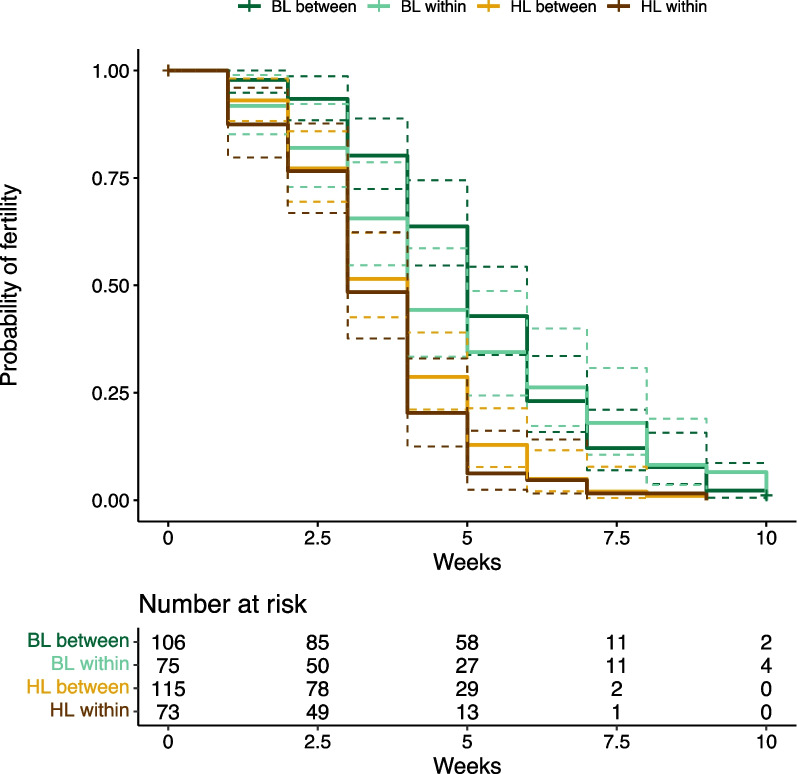
The onset of infertility including the risk table for all four lineage crosses. Survival curves are represented by solid lines, whereas dashed lines show 95% confidence intervals. The dark green line and bright brown line show between-lineage crosses, BL x BL and HL x HL, respectively. The bright green line and dark brown line show within-lineage crosses, BL x HL and HL x BL, respectively

The interaction lineage cross type x female lineage (Mixed effects Cox model: X^2^ = 0.152, df = 1, *P* = 0.696 (Fig. 2) or the effect of lineage cross type (Mixed effects Cox model: X^2^ = 0.659, df = 1, *P* = 0.417) were not significant. HL females started to lay unfertilized eggs significantly earlier than BL females (Mixed effects Cox model: X^2^ = 34.781, df = 1, *P* < 0.0001).

Female survival was similar between lineage cross types (Mixed effects Cox model: lineage cross type: X^2^ = 2.410, df = 1, *P* = 0.121), which was independent of the female lineage (Mixed effects Cox model: female lineage x lineage cross type: X^2^ = 0.316, df = 1, *P* = 0.574). Over the first twenty weeks, more HL than BL females perished; after that, survival probability was similar (Mixed effects Cox model: X^2^ = 7.823, df = 1, *P* = 0.005) (Additional file 1: Fig. S3).

**Fig. 3:**
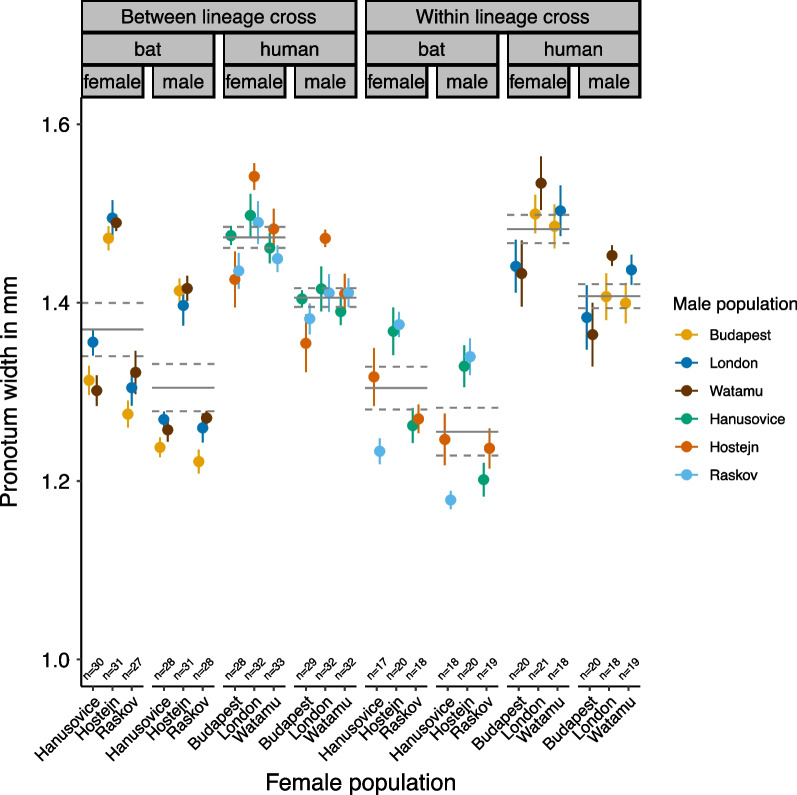
The offspring size as the pronotum width in mm for each population cross. The coloured symbols show population means and the lines show means for lineage crosses with the dashed lines representing one standard error. The female populations are denoted on the x-axis and the colors represent the male populations. The error bars represent one standard error

Offspring size was significantly positively related to the size of the mother (LME: F_1,281.11_ = 11.738, *P* < 0.001). This result remained the same when we accounted for the number of male and female offspring measured in each cross by comparing mean offspring size (LME with average female and male offspring size per mating pair: F_1,280.17_ = 12.280, *P* < 0.001). However, the three-way interaction of offspring sex x lineage cross type x female lineage was significant (LME: F_1,2547.13_ = 11.913, *P* < 0.001) (Fig. 3): Size differences between female and male offspring were smaller in BL crosses than in HL crosses. Also, the HL between-lineage crosses were smaller than HL within-lineage crosses, which was the other way around for BL between- and within-lineage crosses. Independent of offspring sex (LME: sex x female lineage: F_1,0.102_ = 2540.31, *P* = 0.750), offspring from BL mothers were smaller than the offspring of HL mothers (LME: F_1,26.83_ = 12.302, *P* < 0.002) (Fig. 3). Female offspring were significantly larger than male offspring across all lineage crosses (LME: F_1,2553.3_ = 231.067, *P* < 0.0001) (Fig. 3). Offspring size differed between lineage cross types depending on offspring sex (LME: sex x lineage cross type: F_1,2552.11_ = 6.404, *P* = 0.011) but independently of the female lineage (LME: female lineage x cross type: F_1,27.07_ = 2.125, *P* = 0.088).

#### Offspring survival

**Fig. 4:**
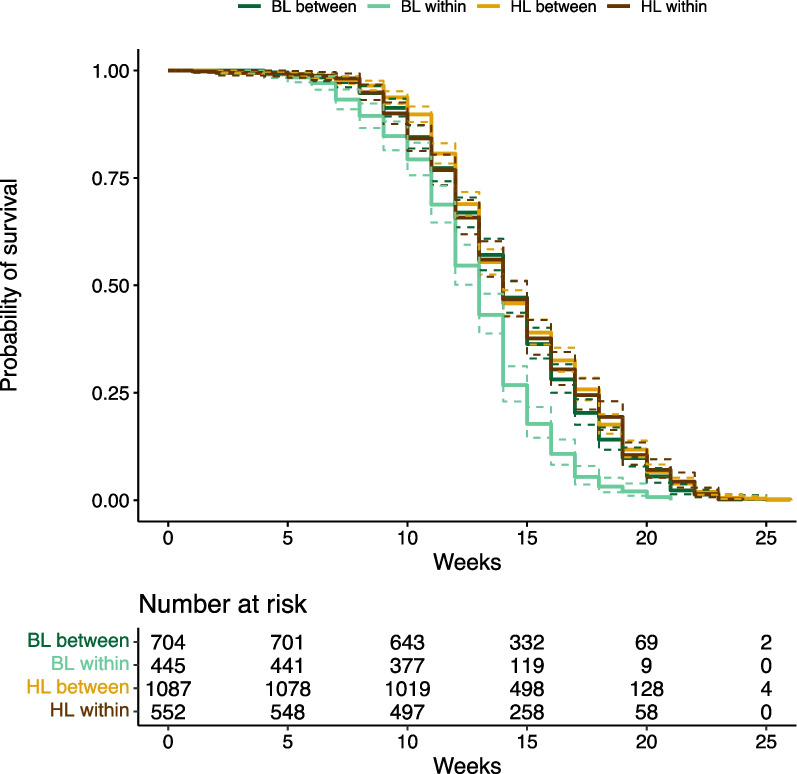
The offspring survival, including the risk table for all four lineage crosses, with both sexes together. The crosses are represented by different colors. Survival curves are represented by solid lines, whereas dashed lines show 95% confidence intervals. The dark green line and bright brown line show between-lineage crosses, BL x HL and HL x BL, respectively. The bright green line and dark brown line show within-lineage crosses, BL x BL and HL x HL, respectively

Overall, offspring survival was the shortest in within-BL crosses and very similar in all other lineage crosses (Fig. 4). When separating for effects of sex and female lineage, we found that offspring survival did not depend on offspring sex, lineage cross type and female lineage (Mixed effects Cox model: sex x female lineage x male lineage: X^2^ = 0.679, df = 1, *P* = 0.410), on offspring sex and cross type (Mixed effects Cox model: X^2^ = 2.701, df = 1, *P* = 0.100), or on lineage cross type and female lineage (Mixed effects Cox model: X^2^ = 0.299, df = 1, *P* = 0.585). Generally, female offspring lived significantly longer than male offspring (Mixed effects Cox model: X^2^ = 196.158, df = 1, *P* < 0.0001) (Additional file 1: Fig. S4). The sex difference in offspring survival was greater in crosses with an HL than a BL mother (Mixed effects Cox model: X^2^ = 73.321, df = 1, *P* < 0.0001) (Additional file 1: Fig. S4).

### Effect of individual populations on fertility

Our results point to a considerable variation in fertility on the population level. Although we did not test these differences statistically, some patterns are rather striking, further illustrating the lack of reproductive isolation between host lineages. For example, the consistent pattern in egg numbers (Fig. 1a) points to a strong effect of the male population on fitness across female populations: females from any of the HL populations laid the most eggs when mated with Hanušovice males, less when mated with Hoštějn males, and the least when mated with Raškov males. In within-BL crosses, Hanušovice males also produced more eggs. This pattern is further supported by the analysis of the weekly egg laying. Both HL and BL females mated with Hanušovice males produced notably higher egg numbers in weeks 5–10 than when mated with males from other populations (Fig. 1b left half; coloured solid lines of HA males exceed the black line of the mean values in the latter half of the period). 

Another example is the effect of Hoštejn females. Out of the three populations used in this study, or out of all nine bat-associated populations that are kept in the Prague laboratory, it prominently shows the largest body size and fertility (unpublished results). Both the body size (in offspring, Fig. 3) and fertility (Fig. 1a) were retained in Hoštejn females relative to other bat-associated populations in both within- and between-lineage crosses.

## Discussion

Our crossing experiment supported those previous studies that showed no reproductive isolation between HL and BL host lineages of the common bedbug. Female fertility-related variables showed very similar values in females mated within or between lineages, with slightly more offspring produced in between-lineage than within-lineage crosses. With HL mothers producing overall more offspring than BL mothers, both in within- and between-lineage crosses, we found a strong female lineage effect.

The offspring fitness parameters showed a slightly different pattern. We found that independently of the lineage cross type, the size difference between females and males was greater in the offspring of HL than BL mothers. Moreover, between-lineage crosses of BL mothers resulted in larger offspring than within-BL crosses, but between- and within-lineage crosses of HL mothers did not show such a difference. A similar trend was evident in adult offspring survival, which was slightly longer in between- than in within-lineage crosses, especially in offspring of BL mothers, but this was not significant (Figs. 3, 4, and Additional file 1: S4). The survival was generally lower in offspring of BL mothers than those of HL mothers.

Higher fertility together with larger, longer living offspring in between- than in within-lineage crosses suggest a heterosis effect could have occurred. However, the between-lineage crosses appeared superior over the within-lineage crosses only in BL. At the same time, BL was shown to lay less eggs and have smaller, shorter living offspring than HL, disregarding the cross type. This might have been caused by different biology of BL or different outcome of laboratory rearing in BL compared to HL. The difference between within and between-lineage crosses in BL would then be caused by a compensation of this difference by mating with HL. Also, if heterosis effects explained this variation, then it would be expected that BL, but not HL, exhibit some degree of inbreeding depression. However, the opposite is more likely to be true, since HL has been shown to be more inbred than BL [22].We also found a considerable effect of individual populations on fertility and offspring fitness, though we did not test this level statistically. The effect was manifested both in females as well as in sperm. Since the effect of an individual population spanned both intra- and inter-lineage crossings, it may be regarded as a further support of gamete compatibility of HL and BL. 

In females, we found that HL females generally laid more eggs than BL females (Fig. 1a). In between-lineage crosses of BL mothers, fitness depended solely on the female genotype, i.e., population. Fitness decreased from Hoštějn to Hanušovice to Raškov mothers independent of the HL father’s genotype. In contrast, in between-lineage crosses of HL mothers, fitness depended on both female and male genotypes (Fig. 1a).

Our results also showed that BL stored sperm for longer than HL. The difference may be due to the observed lower egg-laying rate and later depletion of sperm. It is also possible that this difference reflects an adaptation of BL to their natural conditions. While HL has a stable blood source throughout the year, BL, at least the central European populations sampled, live in seasonal maternity bat colonies that leave the roosts for each winter. BL colonies may therefore suffer from frequent bottlenecks because they overwinter in summer bat roosts without bats being present. Long sperm storage may thus enable surviving females to produce offspring and re-establish the population without males when the host returns in spring. Such an ability has been previously shown in *Cimex vicarius* [40].

Our results on reproductive isolation agree with those of DeVries et al. [31, 32] and Křemenová et al. [30], but are in contrast with those of Wawrocka et al. [29]. The earlier results by Wawrocka et al*.* [29] were produced using BL lineages collected in bat roosts and subsequently reared on human blood for only one generation, or even less, before entering the crossing experiments with HL. The incompatibility shown by Wawrocka et al. [29] could, therefore, have been caused by exposure to a novel diet. DeVries et al. [31] argued that the generally low fecundity of the crosses by Wawrocka et al. [29] could indicate an insufficient adaptation of the BL populations to lab conditions. In our study, we circumvented this by exposing all populations to the novel diet for two years prior to the beginning of the study. The results by Wawrocka et al. [29] may also represent a carryover effect of the environment or parental diet (bat blood) on the sperm or female physiology of the BL populations used. Environmental effects acting on sperm function and their compatibility with the female environment during reproduction are well known [41]. For bedbugs, Křemenová et al. [30] showed this to be true for diet in general, but diet did not appear to affect the HL and BL gamete compatibility, although this was investigated only in one direction using HL females.

Given the lack of post-mating reproductive isolation between BL and HL, the clear genetic differentiation of these lineages needs to be explained by other mechanisms. Pre-copulatory mechanisms are one candidate, although they are more likely to evolve if there are post-copulatory barriers [42]. The divergence between HL and BL in semiochemicals has been studied in the context of aggregation, with no specific preferences identified [31, 43]. Whether the semiochemicals affect the olfactory communication and behaviour in between-lineage mating remains to be investigated.

It is also possible that the synanthropy of BL and HL does not allow for contact frequent enough for genetic exchange. While *Cimex pipistrelli* Jenyns, a strictly bat-associated species, is often found to penetrate homes due to the behavior of host bat species of the genus *Nyctalus* [44], *Cimex lectularius* is extremely rare among *Nyctalus* bats [24], and the behaviour of the main known host species*, Myotis myotis*, does not lead to frequent bedbug dispersal [45]. Given these considerations, it seems likely that the highly inbred population structure [22, 39, 46] is driving bedbug populations apart genetically due to genetic drift, not leading to reproduction isolation yet.

## Conclusions

Despite surpassing previous studies in scope and sample size, we did not find post-mating reproductive barriers necessary to explain the genetic divergence between bat- and human-associated bedbug lineages. We have shown clear differences between the lineages in terms of female fecundity and offspring fitness, but neither these differences nor the potential of genetic drift to drive populations apart can solve the enigma of systematic genetic differences between lineages. The full post-mating compatibility between lineages suggests that any existing pre-mating barriers must be strong and remain to be investigated as a particularly significant case of host-driven differentiation of populations.

## Material and methods

The effect of genetic differentiation on reproductive isolation is assessed by measuring gametic compatibility and hybrid fitness using three population replicates for both HL and BL.

### Populations and rearing conditions

We used three human-associated populations (London, UK, collected and introduced to culture in 2008; Budapest, Hungary, 2010; Watamu, Kenya, 2010) and three bat-associated populations (Hanušovice, CZ, 2016; Hoštějn, CZ, 2016; Raškov, CZ, 2016). The human-associated population replicates were chosen with respect to the large distances of their places of origin. The choice of the bat-related populations was limited to those well habituated to the artificial feeding system, where only populations from Czech Republic were available. All populations were reared in an incubator at 27 °C (optimal temperature to maintain weekly interval for feeding, egg laying, molting etc., according to our experience, or e.g. 26,32,43), at 70% relative humidity with a daily cycle of 12L: 12D. The populations as well as the experimental females and their offspring were artificially fed on a blood source that was novel for both HL and BL. The novelty is assumed based on a lipidomic analysis of bat blood, human blood, human blood conserved in CPDA (citrate phosphate dextrose adenine, Faculty Hospital Bohunice, Brno), or sperm of bedbugs fed with either of the three blood types showed clearly distinct profiles [47] [Additional file 1: Figure S5]. The CPDA-conserved blood was fed using parafilm bags and artificial feeding system [48]. All populations had been habituated to the feeding system for at least two years prior to the experiment.

### Design of the crossing experiments

Bed bugs entering the experiments were virgin. This was achieved by separating fed 5th instars individually into 96-well microplate wells, letting them molt into adults. Three-week-old females from each population were individually single-mated with a three-week-old male. The male came from any of the other five populations, that is, three between-lineage and two within-lineage crosses (N = 369; for sample sizes in individual lineage crosses, see Table 1). Within-population crosses were not executed to avoid confounding effects of inbreeding [39]. The crosses were conducted in four batches across 18 months, all with approximately equal numbers of population cross combinations. The Raškov population was not available for the first batch.

**Table 1:** Crossing scheme and numbers of females in each population cross

Sex			Female					
	Host lineage		Human (HL)			Bat (BL)		
		Population	F4	H1	K17	HA	HO	RA
Male	Human (HL)	F4	–	12	11	11	13	12
		H1	12	–	12	12	11	11
		K17	13	13	–	12	10	14
	Bat (BL)	HA	18	12	12	–	13	13
		HO	14	13	12	14	–	13
		RA	12	11	11	11	11	–

The adult females were fed twice and mated immediately after the second blood meal. Males were fed twice with the second blood meal administered a week before mating. In this way, we ensured full sperm vesicles and males’ eagerness to mate [35]. To ensure that the amount of sperm injected was similar, mating was standardized by interrupting after 60 s after successful intromission [49].

After mating, the females were isolated in a vial equipped with filter paper for egg laying. Females were fed weekly. We recorded if the females fed successfully and counted the number of eggs every week. To measure female fertility, fertilized and unfertilized eggs were distinguished, and the onset of infertility was determined following Otti et al. [34]: fertile eggs are taut and whitish, with visible red eye spots of the developing embryo. Unfertilized eggs normally collapse soon after being laid and are greyish. The onset of infertility was established as the time point when an unfertilized egg was laid for the second time, to allow for one accidental fertilization failure. The total number of fertilized eggs was used to investigate the fecundity of the within- and between-lineage crosses.

If a female stopped laying eggs for two weeks in a row, we placed it in a well of a ventilated 96-well microplate. We recorded its survival in weeks and, since interspecific mating can be harmful in bedbug species [50, 51], female lifespan was used as an additional measure of the male effect on females. In total, we analyzed 369 females in 30 population combinations.

In order to analyze offspring fitness-related traits, the survival and body size, we collected 10–12 fed fifth instar nymphs from each female and transferred them to individual wells of 96-well microplates. This way we aimed to yield at least three sons and three daughters per female. After eclosion to adulthood, we recorded their survival without access to food. After the females and offspring perished, we measured their pronotum width as a representative scale of body size [21]. In total, we analyzed 2791 offspring of 305 females.

### Statistical analyses

Statistical analyses were carried out using RStudio 1.4.1717 (R version 4.1.1, [52]), [51], with packages *lme4* [53], *lmerTest* [54], and *coxme* [55]. First, we performed a Fisher exact test to investigate whether the number of females that did not lay any eggs differed between the lineage crosses (19 females did not lay any eggs: 5 females BL x BL (female x male lineage), 7 females BL x HL, 5 females HL x BL, 2 females HL x HL). Then we tested whether laying no eggs and the number of females dying during the egg-laying period differed between the lineage crosses (51 females perished during egg-laying: 7 females BL x BL, 8 females BL x HL, 23 females HL x BL, 13 females HL x HL). We analysed the first ten weeks of egg-laying because by then almost 95% of the females had stopped laying fertilized eggs (347 out of 369).

To analyze the total number and the number of fertilized eggs, we fitted linear mixed effects models (LME) with lineage cross type (between- x within-lineage cross) and female lineage (bat- x human-associated) including their interaction term as fixed factors and population cross (female x male population) and batch as random effects. Because females occasionally failed to feed in every week, the number of feedings varied between females. Therefore, we fitted the total number of feedings as a covariate in all models. Both fertilized and total number of eggs were significantly positively related to the number of times a female fed over the egg-laying period (LME: fertilized eggs—F_1,354.59_ = 61.970, *P* < 0.0001; total eggs: F_1,354.56_ = 95.746, *P* < 0.0001) (see also the supplementary results in Additional file 1).

For fertilized eggs, we also investigated whether egg-laying patterns differed between lineages by fitting an LME with fertilized eggs laid in each week as a response variable. The week, lineage cross type, female lineage, and their interaction terms were fitted as fixed factors. Using a binary variable of whether a female fed in a particular week, we accounted for variation in feeding behavior among females. Finally, we fitted individual, population cross, and batch as random effects.

For the analysis of the proportion of unfertilized eggs, we used the *cbind()* function in R to combine the number of fertilized and unfertilized eggs as a response variable. With this response variable, we then fitted a generalized linear mixed-effects model (GLME) with binomial distribution. Further, the lineage cross type, female lineage and their interaction term were fitted as fixed factors and the population cross and batch as random effects. Overdispersion was investigated using the *DHARMa* package [56]. If overdispersion was detected, we accounted for it using an object-level random effect.

The *coxme* [55] and *multcomp* [57] packages were used to analyze the onset of infertility, female survival, and the survival of offspring. The lineage cross type, female lineage, and their interaction were fitted as a fixed factor, and the population cross and batch were fitted as random effects. Again, we fitted the number of feedings over the egg-laying period as a covariate. For the survival analysis of offspring (F1 adults), we additionally fitted sex and its interaction with the lineage cross type and the female lineage as fixed factors, and the population cross and mating pair were fitted as random effects. Because the offspring size could affect survival, we fitted the pronotum width as a covariate.

We analyzed adult offspring size, i.e., pronotum width, of the different populations fitting LMEs with female (mother) pronotum width, lineage cross type, female lineage, and offspring sex including their interaction terms as fixed factors, and population cross and mating pair as random effects.

### Microsatellite genotyping

We tested the independence and inbreeding levels of populations used in the study using a set of 9 microsatellite markers [39] (for the primer and multiplexing details, see Additional file 2: Table S5; for the PCR protocol, see Additional file 2: Table S6). We sampled 24–56 individuals from each population and extracted their DNA using PCRBioRapid kit (PB10.24, PCRBiosystems, London, UK). The fragment analysis was carried out at the Genetics Facility at the University of Bayreuth, using the Fragment Analyzer 5200 (Agilent Technologies, Waldbronn, Germany). Alleles were scored using PROSize version 3.0 [58]. For the microsatellite data, we checked for the presence of null alleles using Micro-checker2.2.3 [59]. The among-population Fst, allele frequencies, heterozygosity indices and AMOVAs (for each locus separately, 1000 permutations) within each host lineage and both lineages together were calculated using Genalex [60].

### Supplementary Information

**Additional file 1**: Supplementary results and figures, detailing on female fecundity and offspring fitness.

**Additional file 2**: Supplementary tables, giving details on the methodology and results of the genetic analyses.

## Data Availability

The datasets generated and analyzed during the current study are available in the Fighare repository, [https://doi.org/10.6084/m9.figshare.22663816].
