# Supplementary material for: Despite genetic isolation in sympatry, post-copulatory reproductive barriers have not evolved between bat- and human-associated common bedbugs (Cimex lectularius L.)
Source: Front Zool. 2023 Nov 10;20:36. doi: 10.1186/s12983-023-00514-y (PMC10636883; doi:10.1186/s12983-023-00514-y)
Supplement: Supplementary file 1 — Additional file 1: Supplementary results and figures, detailing on female fecundity and offspring fitness. [file 12983_2023_514_MOESM1_ESM.docx]

**Additional file 1**

**Supplementary results and figures for the article by Sasínková et al. named “Despite genetic isolation in sympatry, post-copulatory reproductive barriers have not evolved between bat- and human-associated common bedbugs (*Cimex lectularius*)”**

**Supplementary results**

**Effect of number of feedings on fecundity**

Both fertilized and total number of eggs were significantly positively related to the number of times a female fed over the egg-laying period (LME: fertilized eggs – F_1,354.59_ = 61.970, P < 0.0001; total eggs: F_1,354.56_ = 95.746, P < 0.0001). Also, feeding had a significant positive effect on the number of fertilized eggs laid per week (LME: F_1,3643.7_ = 110.502, P < 0.0001)**.** The proportion of unfertilized eggs was significantly smaller the more feedings a female took over the egg-laying period (GLME with binomial distribution: X^2^ = 44.630, df = 1, P < 0.0001). However, the onset of infertility was not affected by female feeding (Mixed effects Cox model: X^2^ = 0.155, df = 1, P = 0.69). Finally, female survival was lower with fewer feedings (Mixed effects Cox model: X^2^ = 49.838, df = 1, P < 0.0001).

**Supplementary figures**

**Figure S1. The total number of eggs laid over nine weeks** for each population cross. Original host of the female population is indicated at the top of the plot. The colored symbols show population means, and the lines show means for lineage crosses with the dashed lines representing one standard error. The female populations are given on the x-axis and male populations are shown with different colors. The error bars represent one standard error.

**Figure S2. The proportion of infertile eggs laid over nine weeks for each population cross.** Original host of the female population is indicated at the top of the plot. The colored symbols show population means, and the lines show means for lineage crosses with the dashed lines representing one standard error. The female populations are given on the x-axis and male populations are shown with different colors. Error bars represent one standard error.

**Figure S3. Female survival for all four lineage crosses.** Solid lines are survival curves, and dashed lines show 95% confidence intervals. A dark green line and bright brown line show between lineage crosses, BL x BL and HL x HL respectively. The bright green line and dark brown line show within lineage crosses, BL x HL and HL x BL respectively.

**Figure S4. Offspring survival** for the between and within lineage crosses and sexes, with males in blue and females in red. Solid lines are survival curves, and dashed lines show 95% confidence intervals.


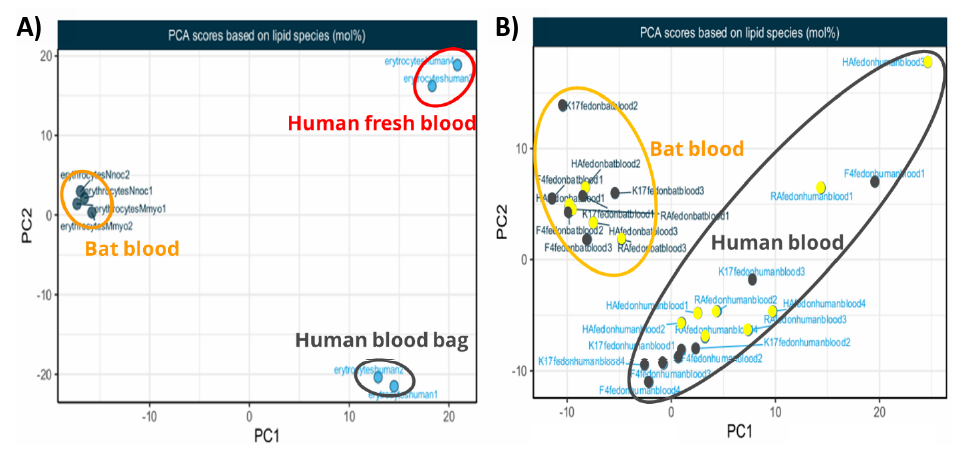


**Figure S5.** **Differences in lipid composition** of erythrocytes between two bed bug hosts (bat and human fresh blood) and within human blood also between fresh and conserved blood (human blood bag). Comparing two bat species: *Nyctalus noctula* (Nnoc) and *Myotis myotis* (Mmyo). **B)** Sperm lipidome in common bed bugs (*Cimex lectularius*) corresponds to the male diet and not to male origin (bat: yellow circles; human: black circles).
