# Supplementary material for: Despite genetic isolation in sympatry, post-copulatory reproductive barriers have not evolved between bat- and human-associated common bedbugs (Cimex lectularius L.)
Source: Front Zool. 2023 Nov 10;20:36. doi: 10.1186/s12983-023-00514-y (PMC10636883; doi:10.1186/s12983-023-00514-y)
Supplement: Supplementary file 2 — Additional file 2: Supplementary tables, giving details on the methodology and results of the genetic analyses. [file 12983_2023_514_MOESM2_ESM.docx]

**Additional file 2**

**Supplementary tables for the article by Sasínková et al. named “Despite genetic isolation in sympatry, post-copulatory reproductive barriers have not evolved between bat- and human-associated common bedbugs (*Cimex lectularius*)”**

**Table S1.** Genetic characteristics of the populations used in the study based on 9 microsatellite loci. Computed using Genalex (2).

| **Population** | **Sample Size** |  | **No. of alleles** | **Observed Heterozygosity** | **Expected Heterozygosity** | **Fixation index (F)** | **No. of private alleles across loci** |
| --- | --- | --- | --- | --- | --- | --- | --- |
| **H1** | 27 | Mean | 3.11 | 0.37 | 0.41 | 0.13 | 11 |
|  |  | SE | 0.48 | 0.09 | 0.08 | 0.11 |  |
| **F4** | 28 | Mean | 2.78 | 0.36 | 0.38 | 0.03 | 7 |
|  |  | SE | 0.40 | 0.09 | 0.08 | 0.11 |  |
| **K17** | 24 | Mean | 3.56 | 0.48 | 0.51 | 0.08 | 13 |
|  |  | SE | 0.71 | 0.09 | 0.07 | 0.10 |  |
| **HO** | 56 | Mean | 3.33 | 0.40 | 0.53 | 0.26 | 10 |
|  |  | SE | 0.24 | 0.07 | 0.02 | 0.12 |  |
| **HA** | 52 | Mean | 3.89 | 0.39 | 0.49 | 0.23 | 5 |
|  |  | SE | 0.26 | 0.08 | 0.06 | 0.09 |  |
| **RA** | 56 | Mean | 4.22 | 0.49 | 0.63 | 0.24 | 7 |
|  |  | SE | 0.43 | 0.08 | 0.03 | 0.09 |  |

**Table S2.** Allelic diversity and heterozygosity at 9 microsatellite loci, including the proportions of putative null alleles. Computed using Genalex (2). HW = p-value indicating the probability of the Hardy-Weinberg equilibrium (*** p < 0.001; ** p < 0.01; * p < 0.05).

| **locus** | **No. of alleles per locus** | **Number of individuals per locus** | **Observed Heterozygosity** | **Expected Heterozygosity** | **Polymorphic information content** | **HW** |
| --- | --- | --- | --- | --- | --- | --- |
| **47A04** | 11 | 203 | 0.552 | 0.826 | 0.804 | *** |
| **BB454_20** | 8 | 219 | 0.183 | 0.830 | 0.807 | *** |
| **48G02** | 11 | 194 | 0.572 | 0.849 | 0.829 | *** |
| **48D02** | 10 | 137 | 0.474 | 0.853 | 0.834 | *** |
| **48E11** | 13 | 217 | 0.373 | 0.852 | 0.833 | *** |
| **47A07** | 10 | 221 | 0.335 | 0.812 | 0.786 | *** |
| **46H08** | 12 | 201 | 0.458 | 0.815 | 0.789 | *** |
| **44G09** | 20 | 215 | 0.684 | 0.829 | 0.814 | *** |
| **BB454_06** | 9 | 217 | 0.129 | 0.713 | 0.660 | *** |

| **Host lineage** |  | Human (HL) | | | Bat (BL) | | |
| --- | --- | --- | --- | --- | --- | --- | --- |
|  | **Population** | **H1** | **F4** | **K17** | **HO** | **HA** | **RA** |
| Human (HL) | **H1** | **0.129** |  |  |  |  |  |
|  | **F4** | 0.464 | **0.034** |  |  |  |  |
|  | **K17** | 0.451 | 0.308 | **0.082** |  |  |  |
| Bat (BL) | **HO** | 0.515 | 0.509 | 0.447 | **0.261** |  |  |
|  | **HA** | 0.536 | 0.509 | 0.457 | 0.401 | **0.232** |  |
|  | **RA** | 0.406 | 0.363 | 0.394 | 0.354 | 0.245 | **0.242** |

**Table S3.** Within population and pairwise Fst. Computed using Genalex (2).

| **Host lineage** |  | Human (HL) | | | Bat (BL) | | |
| --- | --- | --- | --- | --- | --- | --- | --- |
|  | **Population** | **H1** | **F4** | **K17** | **HO** | **HA** | **RA** |
| Human (HL) | **H1** | **10.650** | 21.704 | 23.228 | 28.542 | 29.170 | 25.815 |
|  | **F4** | 21.704 | **12.812** | 19.176 | 27.940 | 27.148 | 23.911 |
|  | **K17** | 23.228 | 19.176 | **12.986** | 26.458 | 26.467 | 26.723 |
| Bat (BL) | **HO** | 28.542 | 27.940 | 26.458 | **16.615** | 25.325 | 25.935 |
|  | **HA** | 29.170 | 27.148 | 26.467 | 25.325 | **16.140** | 22.396 |
|  | **RA** | 25.815 | 23.911 | 26.723 | 25.935 | 22.396 | **18.823** |

**Table S4.** Pairwise population matrix of mean among population Nei´s genetic distance. Computed using Genalex (2).

**Table S5.** Description of microsatellite primers (following (1) arranged in 3 multiplex panels. T_a_ = annealing temperature (^o^C).

| **Locus** | **Sequence (5’–3’)** | **Multiplex** | **T_a_** | **Expected/Observed size (bp)** |
| --- | --- | --- | --- | --- |
| 47A04 | F: CCATTGACGGAGGGTTGCTTC | 1 | 60 | 164 |
|  | R: CACTTTCTTGTAACCATCACCATC |  |  | 149–227 |
| BB454_20 | F: GCAACCCTGGACTTCTCAAC | 1 | 60 | 188 |
|  | R: TCAGCTCTCCATTAGAACGAAAC |  |  | 235–267 |
| 48G02 | F: TCATATGGGCGGATTAGAGC | 1 | 60 | 302 |
|  | R: TAACAATCTGGAGGCGGAAC |  |  | 292–364 |
| 48D02 | F: AAATAATTTAGCTGCAAACAATAGG | 2 | 60 | 182 |
|  | R: TTGCCAGATTTCTCAATCG |  |  | 146–208 |
| 48E11 | F: TTCGTTTGTGTAGAACCTTGG | 2 | 60 | 269 |
|  | R: TACGTCCCTACAAGCTCACC |  |  | 218–276 |
| 47A07 | F: AGATAGGGCAACCTTTCAGAG | 2 | 60 | 315 |
|  | R: TTGGTGATAGTGAACGAACG |  |  | 298–355 |
| 46H08 | F: TTGTGAGTGTGTCTCTCTCTACTGTG | 3 | 60 | 144 |
|  | R: CAGGTTCACAGGCCAAATG |  |  | 139–165 |
| BB454_06 | F: TCGTGTCCACGCCTTAAAC | 3 | 60 | 288 |
|  | R: ATTCGTATACTCTCTCGAATTCTGC |  |  | 271–303 |
| 44G09 | F: TTCACAGATTTAAGCCTAACTGGTC | 4 | 60 | 233 |
|  | R: CAAATAACCTCGAATTCATACGC |  |  | 181–282 |

**Table S6.** PCR protocol for amplification of the microsatellite loci.

| **Step** | **Temperature (°C)** | **Duration (min)** | **Cycles** |
| --- | --- | --- | --- |
| Initial Denaturation | 95 | 15:00 | 1 |
| Subsequent Denaturation | 94 | 0:30 | 30 |
| Annealing | 60 | 1:30 |  |
| Extension | 72 | 1:00 |  |
| Final Elongation | 60 | 30:00 | 1 |

**References:**

1. Fountain T, Duvaux L, Horsburgh G, Reinhardt K, Butlin RK. Human-facilitated metapopulation dynamics in an emerging pest species, *Cimex lectularius*. Mol Ecol. březen 2014;23(5):1071–84.

2. Peakall R, Smouse P. GENALEX 6: genetic analysis in Excel. Population genetic software for teaching and research. Mol Ecol Notes. 2006;6:288–95.
